# The diagnosis of DIC: a current overview

**DOI:** 10.3389/fmed.2025.1502628

**Published:** 2025-01-31

**Authors:** Hongyu Yang, Xiaochun Ma, Xu Li

**Affiliations:** Department of Critical Care Medicine, The First Affiliated Hospital, China Medical University, Shenyang, Liaoning, China

**Keywords:** disseminated intravascular coagulation, diagnostic criteria, coagulation, fibrinolysis, platelet

## Abstract

The name of disseminated intravascular coagulation (DIC) and its diagnostic criteria remain controversial. DIC is a clinical syndrome caused by a variety of etiologies, which determines its high heterogeneity. It is inappropriate to adopt the same diagnostic criteria. DIC has its common characteristics. First, in most DIC, thrombosis and bleeding coexist. Second, DIC is a dynamic process. Third, endothelial cell injury and systemic coagulation activation are the core of DIC. Fourth, DIC is an initiating factor of multiple organ dysfunction syndrome (MODS). There are still controversies about the diagnostic criteria of DIC. First, it relies on clinical manifestations and laboratory tests, which cannot reflect pathophysiology. Second, the clinical manifestations were not sensitive or specific. Third, there is a lack of sensitive biomarkers. Fourth, the parameters in the current diagnostic criteria cannot fully reflect the actual coagulation function. Fifth, it is obviously inappropriate to use the same scoring system for diagnosis of clinical syndromes with different etiologies and pathophysiology. Therefore, it is urgent to re-establish the diagnostic criteria for DIC. In recent years, the understanding of DIC has been continuously improved, including the in-depth understanding of the pathogenesis, the classification of coagulation phenotypes, and the development of the “two-step” diagnosis of DIC, etc. All of these contribute to the establishment of new diagnostic criteria for DIC. In conclusion, it is necessary to develop personalized diagnostic criteria based on etiology, reflecting pathophysiological mechanisms, establishing clear cut-off values for parameters, being clinical applicable, being globally unified, and most importantly, being able to identify therapeutic targets.

## 1 Introduction

Disseminated intravascular coagulation (DIC) has been variously named since it was reported, including “disseminated international confusion”, “death is coming”, “consumption coagulopathy”, “defibrination syndrome”, “diffuse intravascular thrombosis”, indicating that its name and severity are still controversial (1). In 2001, the International Society on Thrombosis and Hemostasis (ISTH) updated the definition of DIC as “an acquired syndrome characterized by the intravascular activation of coagulation with loss of localization arising from different causes. It can originate from and cause damage to the microvasculature, which if sufficiently severe, can produce organ dysfunction” (2). Compared to the traditional DIC definition, the new definition has three changes: First, it does not emphasize fibrinolysis as a necessary condition for DIC, because fibrinolysis is usually secondary, and most DIC does not occur fibrinolysis in the initial stage. Second, DIC is not emphasized as an acquired bleeding syndrome, because thrombus and bleeding coexist in most cases. Third, DIC mainly involves the microvascular system and causes each other. That is, microcirculatory endothelial cell injury leads to DIC, and the occurrence of DIC further aggravates microcirculatory dysfunction. DIC is the end-stage manifestation of many diseases and widely exists in various clinical departments, so it is necessary to detect DIC early. The diagnosis of DIC has been widely debated over the years, so it is necessary to summarize its development and progress. DIC is divided into acute and chronic according to the course. We’ll discuss acute DIC because of its severe consequences.

## 2 The pathophysiology of DIC

**FIGURE 1:**
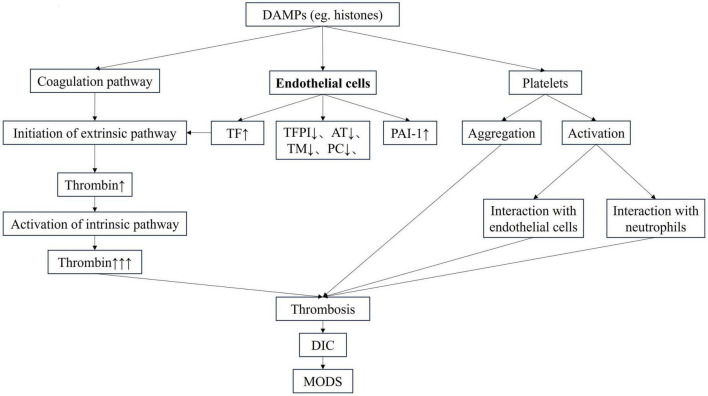
The pathophysiology of DIC. AT, antithrombin; DAMP, damage-associated molecular pattern; DIC, disseminated intravascular coagulation; MODS, multiple organ dysfunction syndrome; PAI-1, plasminogen activator inhibitor-1; PC, protein C; TF, tissue factor; TFPI, tissue factor pathway inhibitor; TM, thrombomodullin.

Disseminated intravascular coagulation is a clinical syndrome caused by the deterioration of coagulation function. The three major factors involved in the coagulation process, including endothelial cells, coagulation factors and platelets are activated and interact with each other (3) (Figure 1). Inflammatory factors damage endothelial cells, aggravate inflammatory response, and further promote the release of inflammatory mediators. At the same time, the expressions of tissue factor (TF) and other pro-coagulant substances are elevated, the physiological anticoagulant pathway is damaged, and fibrinolytic inhibitors are increased, which jointly lead to coagulation activation and microvascular thrombosis. Inflammation interacts with the coagulation system, forming a vicious cycle (4, 5). Endothelial cells injury and systemic coagulation activation are the central pathophysiological changes in DIC, and are also important features that distinguish DIC from other severe coagulation changes (3). Emerging biomarkers, such as histones and angiopoietin-2 (Ang-2), have great potential in the diagnosis of DIC, especially in sepsis. As important DAMPs, histones are released in sepsis and mediate endothelial cell damage (6). Recent studies have shown that histones promote coagulation activation and even DIC in sepsis, leading to the occurrence of multiple organ dysfunction syndrome (MODS) and even death (7, 8). Recent studies have found that Ang-2 plays a central role in the process of sepsis. It is a core molecule linking vascular function, inflammation, coagulation activation and complement system (9). Ang-2 is positively correlated with the risk of death in sepsis patients and may serve as a useful and valuable biomarker for predicting mortality in septic adult patients (10).

## 3 The common characteristics of DIC

The clinical manifestations of DIC are complex, but there are also common features.

### 3.1 Different etiologies determine the heterogeneity of DIC

Disseminated intravascular coagulation is caused by multiple causes, with different pathophysiological mechanisms and diverse clinical manifestations. Severe infection, trauma, pathological obstetrics, malignant tumor and poisoning, etc., can all lead to coagulation dysfunction and gradually progress to DIC (11). Therefore, DIC is the result of a deterioration in coagulation function. In the process of coagulation activation caused by severe infection, endothelial cells change from anticoagulant surface to pro-coagulant surface, the production and release of coagulation factors increase, and platelets are activated, resulting in hypercoagulable state and microvascular thrombosis, which further affects tissue perfusion and leads to organ dysfunction. Therefore, it is thrombotic DIC (12, 13). However, DIC caused by trauma is due to loss and dilution of hemostatic substrates such as coagulation factors and platelets, as well as the activation of fibrinolysis, so the clinical manifestation is bleeding type (13, 14). Therefore, DIC, similar to ARDS and sepsis, is not a disease, but a clinical syndrome. The clinical manifestations of DIC caused by different etiologies vary greatly, so the treatments are also different. Therefore, it is necessary to establish new diagnostic criteria according to the pathophysiological changes of DIC.

### 3.2 Coexistence of thrombus and bleeding

The clinical manifestations of DIC, including hypercoagulation and hypocoagulation, thrombus and bleeding, are not clearly defined and often coexist (11). For example, in the lipopolysaccharide (LPS)-induced rat model of sepsis, bleeding was observed at 10 min after LPS injection. The bleeding area expanded at 30 min. Thrombus was formed in the microvessel at 60 min, and thrombosis aggravated at 120 min, resulting in the cessation of blood flow in the distal side of the vessel, indicating that thrombosis and bleeding coexisted (15). The same is true in the rat model of trauma, the microthrombus was seen in the mesenteric venule at 30 min. The thrombus dissolved at 180 min. However, extravasation of red and white blood cells (micro bleeding) increased (3). The causes of DIC determines the clinical manifestations, some are mainly bleeding, some are mainly thrombosis, but in most cases both exist simultaneously. However, there is no direct evidence of microthrombosis, which is easy to be ignored clinically (16).

### 3.3 The dynamic process in DIC

Hypercoagulation and hypocoagulation, coagulation and fibrinolysis, compensation and decompensation evolve dynamically in DIC. In trauma-induced DIC, thrombin production is increased due to post-traumatic tissue damage and bleeding, leading to hypercoagulation. With the progression of disease and the increase of blood loss, the endothelial cell damage worsens and it changes to hypocoagulation. With the gradual cessation of bleeding, combined with the supplementation of clotting substrates, and the inhibition of fibrinolysis by the production of thrombin-induced plasminogen activator inhibitor-1 (PAI-1), which leads to the conversion to hypercoagulation. It reflects the evolution between hypercoagulation and hypocoagulation, and between coagulation and fibrinolysis (17). In sepsis-induced DIC, the invasion of pathogen is first recognized by the innate immune system, leading to activation of endothelial cells and immune cells to form local clots that prevent the spread of the pathogen, known as “immunothrombosis” (18, 19). At this point, the body’s coagulation function is compensatory. As the disease progresses, coagulation activation is further aggravated, leading to widespread thrombosis in the microcirculation, tissue ischemia and hypoxia, and then organ dysfunction. This is a decompensated state (12, 20, 21).

### 3.4 Endothelial cells injury as the core of DIC

The central pathophysiological changes of DIC caused by various etiologies are endothelial cells injury and systemic coagulation activation, which reflects the characteristics of “disseminated” (22). This is what distinguishes it from local thrombotic disease.

### 3.5 DIC as an initiating factor of MODS

Endothelial cells line the luminal surface of all blood vessels, and are therefore the first barrier that separates blood and tissue. Endothelial cell injury promotes coagulation activation and DIC. Loss of homeostasis of the coagulation system leads to microthrombosis, tissue hypoperfusion and organ dysfunction. Thus, DIC is considered to be an initiating factor of MODS rather than one of the organs (23).

## 4 The diagnostic criteria of DIC and the existing controversies

**FIGURE 2:**
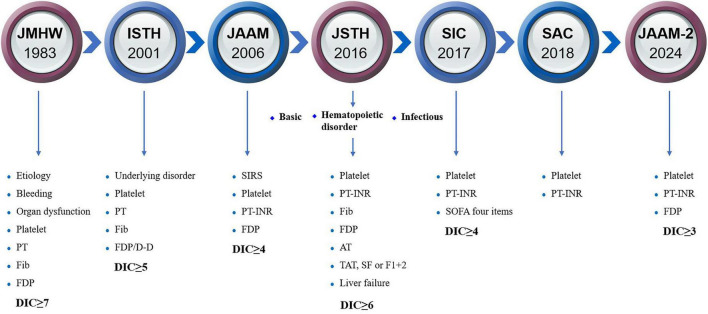
The history of diagnostic criteria for DIC. AT, antithrombin; D-D, D-dimer; DIC, disseminated intravascular coagulation; F1 + 2, prothrombin fragment 1 + 2; FDP, fibrinogen degradation product; Fib, fibrinogen; ISTH, International Society on Thrombosis and Hemostasis; JAAM, Japanese Association for Acute Medicine; JMHW, Japanese Ministry of Health and Welfare; JSTH, Japanese Society on Thrombosis and Hemostasis; PT-INR, prothrombin time-international normalized ratio; SAC, sepsis-associated coagulopathy; SF, soluble fibrin; SIC, sepsis-induced coagulopathy; SIRS, systemic inflammatory response syndrome; SOFA, sequential organ failure assessment; TAT, thrombin-antithrombin.

There is no gold standard for the diagnosis of DIC, which is currently based on the DIC scoring system (Figure 2), including Japanese Ministry of Health and Welfare (JMHW) criteria in 1983 (24), ISTH criteria in 2001 (2), Japanese Association for Acute Medicine (JAAM) criteria in 2006 (25), Japanese Society on Thrombosis and Hemostasis (JSTH) criteria in 2016 (26), sepsis-induced coagulopathy (SIC) criteria in 2017 (27) (Table 1), sepsis-associated coagulopathy (SAC) criteria in 2018 (28) (Table 2) and JAAM-2 criteria in 2024 (29) (Table 1). The two most commonly used criteria are ISTH and JAAM, both of which integrate multiple clinical and laboratory parameters. Both criteria have advantages and disadvantages (Table 3), and neither can be widely applied to DIC caused by various etiologies. JSTH criteria in 2016 was developed according to different causes such as hematopoietic disorders and infection. However, too many parameters were included, and the biomarkers were not widely tested, thus affecting its clinical application. Even though a simplified JSTH scoring system was developed in 2017 (30), including only platelets, prothrombin time-international normalized ration (PT-INR), fibrinogen degradation product (FDP) and antithrombin (AT), it is still not widely accepted. The diagnostic criteria for sepsis-induced coagulation dysfunction proposed in 2017 and 2018 were SIC and SAC, respectively. Compared with overt-ISTH and JAAM criteria, JAAM and SIC criteria were more sensitive and less specific; the parameters included in SIC and SAC criteria were relatively simple and convenient for clinical application (31). However, the combination of JAAM score or overt-ISTH score with SIC score did not improve the sensitivity and specificity compared to the application alone. There were overlaps among patients diagnosed according to all four criteria (32).

**TABLE 1:** Disseminated intravascular coagulation (DIC) scoring systems.

	Score	overt-ISTH	JAAM	SIC	JAAM-2
Year	–	2001	2006	2017	2024
SIRS score	1	–	≥3	–	–
Platelet count (× 10^9^/L)	1	≥50, <100	≥80, <120 or ≥30% decrease within 24 h	≥100, <150	≥80, <120 or ≥30% decrease within 24 h
	2	<50	–	<100	–
	3		<80 or ≥50% decrease within 24 h		<80 or ≥50% decrease within 24 h
PT ratio	1	> 3 ∼ ≤ 6	PT-INR ≥ 1.2	1.2 < PT-INR ≤ 1.4	PT-INR ≥ 1.2
	2	>6	–	PT-INR > 1.4	–
Fib (g/L)	1	<1.0	–	–	–
	2	–	–	–	–
FDP (μg/ml)/D-D	1	–	FDP ≥ 10, < 25	–	FDP ≥ 10, < 25
	2	Moderate increase	–	–	–
	3	Strong increase	FDP > 25	–	FDP > 25
SOFA four items score*	1	–	–	1	–
	2	–	–	≥ 2	–
Total score for DIC or SIC		≥ 5	≥4	≥4	≥ 3

*Respiratory SOFA, cardiovascular SOFA, hepatic SOFA, renal SOFA. D-D, D-dimer; DIC, disseminated intravascular coagulation; FDP, fibrinogen degradation product; Fib, fibrinogen; ISTH, International Society on Thrombosis and Hemostasis; JAAM, Japanese Association for Acute Medicine; PT-INR, prothrombin time-international normalized ratio; SIC, sepsis-induced coagulopathy; SIRS, systemic inflammatory response syndrome; SOFA, sequential organ failure assessment.

**TABLE 2:** Sepsis-associated coagulopathy (SAC) scoring system.

		Platelet count (× 10^9^/L)			
		**≥150**	**100∼150**	**80∼100**	**< 80**
PT-INR	<1.2	No SAC	No SAC	No SAC	No SAC
	1.2 ∼ 1.4	No SAC	Mild	Moderate	Moderate
	1.4 ∼ 1.6	No SAC	Moderate	Moderate	Moderate
	≥ 1.6	No SAC	Moderate	Moderate	Severe

PT-INR, prothrombin time-international normalized ratio; SAC, sepsis-associated coagulopathy.

**TABLE 3:** Advantages and disadvantages of International Society on Thrombosis and Hemostasis (ISTH) criteria and Japanese Association for Acute Medicine (JAAM) criteria.

DIC scoring systems	Advantages	Disadvantages
ISTH	1. Can be used for critically ill adults and children.	1. Diagnosis of non-overt DIC requires hematologic markers, which are not widely available.
	2. Suitable for both infectious and non-infective DIC.	2. Contains Fib, which has limited value in sepsis as an acute reaction protein.
	3. Distinguish between overt and non-overt DIC. The patients with non-overt DIC can be treated.	3. FDP or D-D has no exact cut-off value, and the subjective influence is obvious.
	4. Involvement of several fibrin-related markers (FDP, D-D), which increased its applicability.	4. The patients who met the criteria for overt-ISTH criteria may already be at late stage, which will delay treatment.
	5. High specificity.	–
JAAM	1. High sensitivity, more suitable for critically ill patients.	1. Lack of evidence in children.
	2. No need to evaluate risk factors.	2. Lack of prospective evaluation of patients with malignant tumors.
	3. Inclusion of SIRS score and thrombocytopenia ratio, which increased the sensitivity to screen for early treatment.	3. Difficult to distinguish survival and death in patients with DIC with low APACHE II scores.
	4. Fib excluded.	4. Moderate specificity.
	5. Earlier diagnosis of DIC than the other two criteria.	5. Sepsis 3.0 definition excluded SIRS score.
	6. Associated with sepsis patients.	–

APACHE II, Acute Physiology and Chronic Health Evaluation II; D-D, D-dimer; DIC, disseminated intravascular coagulation; FDP, fibrinogen degradation product; Fib, fibrinogen; ISTH, International Society on Thrombosis and Hemostasis; JAAM, Japanese Association for Acute Medicine; SIRS, systemic inflammatory response syndrome.

Therefore, it seems that the current DIC diagnostic criteria cannot meet the *status quo*, and there is still a large room for improvement.

### 4.1 The necessity of differential diagnosis based on DIC score

The current diagnostic criteria rely solely on clinical features and laboratory test results, and cannot reflect pathophysiological changes, especially endothelial cells damage (33). The differential diagnosis of acute DIC is very important, and it may be difficult to distinguish it from other diseases by clinical and laboratory parameters alone. For example, heparin-induced thrombocytopenia (HIT) may cause thrombosis and thrombocytopenia. Thrombotic thrombocytopenic purpura (TTP) leads to thrombosis, organ dysfunction and thrombocytopenia. Antiphospholipid syndrome (APS) can result in thrombosis, thrombocytopenia and prolonged activated partial thromboplastin time (APTT). Severe form of APS may lead to rapid onset of multiple organ dysfunction. Liver cirrhosis can cause bleeding tendency, prolonged PT, prolonged APTT and thrombocytopenia. All of the above may meet the current DIC diagnostic criteria, but they are not DIC and need to be differentiated (34, 35). Early local bleeding in patients with aortic aneurysm leads to prolonged PT, increased D-dimer (D-D), and thrombocytopenia, but coagulation activation is localized without systemic endothelial cell damage. It mimics DIC, but is not DIC (3). Therefore, the current DIC diagnostic criteria that rely solely on clinical manifestations and laboratory findings are not appropriate.

### 4.2 Atypical clinical manifestations of DIC

Clinical features, as parameters of diagnostic criteria, cannot fully reflect the actual situation. The signs of fulminant purpura or hemorrhagic embolism, or hemodynamic instability resulting from large blood vessel embolism may be evaluated clinically. However, the microcirculatory embolism is not easy to detect clinically and may delay diagnosis, especially in sepsis-induced DIC.

### 4.3 Lack of sensitive biomarkers

Diagnosis is for proper treatment, so the diagnostic criteria need to be highly sensitive and specific. At present, the diagnostic criteria rely on laboratory test results, which are not sensitive. Many studies have explored biomarkers for early prediction of coagulation dysfunction, most of which are related to endothelial cell damage (8, 10, 36). However, multiple systems such as coagulation and inflammation interact, so it is key to include biomarkers that reflect multi-system changes in DIC diagnostic criteria (37). Currently, most biomarkers are not widely tested clinically, limiting their inclusion in the diagnostic criteria.

### 4.4 Complexity of DIC as a clinical syndrome

Disseminated intravascular coagulation caused by different etiologies has different pathophysiology, so different diagnostic criteria should be adopted. Sepsis, acute respiratory distress syndrome (ARDS) and DIC are all clinical syndromes with great heterogeneity and need to be phenotyped in order to adopt similar treatments for patients with the same pathophysiology (38, 39). At present, there have been some attempts to classify DIC, such as “coagulation phenotype” and “fibrinolytic phenotype” according to coagulation or fibrinolytic changes. The former is represented by sepsis, which is mainly characterized by thrombosis, tissue hypoperfusion and organ dysfunction. Coagulation activation and suppressed fibrinolysis are common features in sepsis. PAI-1 is significantly increased, and fibrinolytic degradation products such as D-D and FDP are slightly elevated. Anticoagulation is the main treatment for this type. DIC with enhanced fibrinolysis can be seen in trauma, and the main clinical manifestations are hemorrhage. It is characterized by hyperfibrinolysis, which manifests as a significant increase in D-D and FDP, and almost no increase in PAI-1. Antifibrinolytic therapy is required in this type. DIC is also divided into “acute” and “chronic” according to the course. There are differences in etiology, course of disease, clinical manifestations, laboratory tests, treatments and prognosis (16, 40). Diseases that cause acute DIC include infection, trauma, pathological obstetrics, etc. Onset is usually within 7 days. The clinical manifestations are mild in the early stage and gradually progress in the middle and late stage. Microcirculation disorders and organ dysfunction are common, and most coagulation tests suggest a decompensated state. The main treatment is to control the etiology and improve coagulation function. The prognosis is poor. Chronic DIC can be seen in malignant tumors, pregnancy process, etc. The course of the disease is usually more than 14 days, and there is no microcirculation disturbance and organ failure. Laboratory tests often reveal a compensatory state. Combined treatment with anticoagulation and antifibrinolysis is effective, and the prognosis is good (3). However, the current classification is not based on pathophysiology, and the personalized diagnostic criteria for DIC based on etiologies are more suitable and helpful.

### 4.5 Lack of thoroughness of DIC parameters

The parameters in the current diagnostic criteria cannot fully reflect the actual coagulation function. At present, most of the DIC diagnostic criteria include coagulation and fibrinolysis parameters and platelet counts, but they cannot represent the overall picture of actual coagulation function. For example, platelet counts are inconsistent with function, and evaluating platelet counts alone is not comprehensive. In addition, platelet count in severe patients is affected by a variety of factors, such as bone marrow suppression, destruction of extracorporeal circulation and immune response, etc., which can lead to thrombocytopenia, but do not represent abnormal coagulation function (41, 42). Fibrinolytic parameters such as D-D and FDP included in the diagnostic criteria cannot fully represent the severity of coagulation dysfunction. For example, fibrinolysis is suppressed in sepsis-induced DIC, so the levels of D-D and FDP underestimate the severity of coagulation activation (43, 44). Fibrinogen (Fib), as an acute reaction protein, is mostly elevated in sepsis and does not reflect changes in coagulation and fibrinolysis function (12). Currently, ISTH is the most commonly used DIC diagnostic criteria in sepsis, which is obviously inappropriate due to the inclusion of Fib.

Personalized DIC diagnostic criteria needs to be re-established according to different causes. No “one-size-fits-all criteria” for DIC!

## 5 The progress made so far

### 5.1 In-depth understanding of pathophysiology

With the in-depth study on the pathophysiology of DIC, it is gradually realized that TF-mediated activation of extrinsic coagulation pathway and the subsequent coagulation dysfunction are the main factors contributing to the progression of DIC (45). In the process of DIC, in addition to coagulation activation, the anticoagulation pathway is also damaged, which aggravates coagulation dysfunction. Interactions between platelets, clotting factors, endothelial cells and neutrophils amplify the coagulation cascade. Therefore, cell-mediated activation of the coagulation system is the basis for initiation, expansion and spread of a series of coagulation cascades (46). Bidirectional regulation of inflammation and coagulation system promotes the onset of DIC (47). Thrombin-antithrombin (TAT) and PAI-1, as key molecules in the pathophysiology of DIC, are altered in the early stage of DIC and may be used as biomarkers for the diagnosis of DIC (33).

### 5.2 Identifying coagulation phenotypes

With the deepening understanding of clinical syndromes such as sepsis and DIC, it has been recognized that the failure of clinical studies on sepsis is probably due to the high heterogeneity of patients, and therefore it is necessary to identify the phenotypes (48, 49). With the development and application of artificial intelligence, Seymour et al. (50) divided sepsis patients into α, β, γ, and δ phenotypes by machine learning method as early as 2019, among which δ phenotype showed the most obvious coagulation changes, mainly manifested by increased TAT, D-D and PAI-1. The mortality rate was also highest in the δ phenotype. In 2021, Kudo et al. (51) divided sepsis patients into dA, dB, dC, and dD phenotypes by machine learning method. Cluster dA had the most severe coagulopathy with high D-D and FDP levels, the most severe organ dysfunction, and the highest mortality. The results of the two studies are similar, suggesting that patients with severe coagulation activation have more organ dysfunction and higher mortality, which is consistent with the previous understanding that “DIC is an initiating factor of MODS.”

### 5.3 Dynamic process of DIC

Disseminated intravascular coagulation is a dynamic process, which requires the diagnostic criteria for the early stage of DIC. In 2019, ISTH proposed that the process from SIC to DIC develops gradually, and DIC caused by sepsis needs to adopt a “two-step” diagnosis scheme (52), that is, sepsis patients with thrombocytopenia should be first screened using SIC scoring system, and early anticoagulant treatments should be carried out for patients meeting the SIC criteria. The overt-ISTH DIC score was evaluated as the second step. DIC patients diagnosed by overt-ISTH criteria should be treated with bundle therapy including anticoagulants. A differential diagnosis should be performed in sepsis patients who do not meet SIC or overt-ISTH criteria. The “two-step” approach represents a continuum of SIC and DIC, where SIC criteria is highly sensitive and more suitable for early screening, followed by early intervention to prevent progression.

## 6 Future perspective

Although “DIC” has been used for many years, its name, diagnostic criteria, and so on are still controversial. For example, DIC is an acronym for “disseminated intravascular coagulation,” but it cannot fully reflect the coagulation dysfunction caused by various etiologies, nor can it represent pathophysiological changes. “Disseminated” reflects the universality of coagulation changes, indicating the involvement of different sites, which may occur in different parts of the same organ or in different organs. However, “intravascular coagulation” has no characteristic clinical signs and can only be indirectly indicated by the occurrence of organ dysfunction or confirmed by autopsy. “Coagulation” stands for coagulation activation, but the same patient is often accompanied by both bleeding and thrombosis clinically, not just “coagulation.” DIC involves many specialties, and the different understanding of pathophysiology among doctors in different specialties is also the reason for the dispute over DIC.

### 6.1 The redefinition of DIC

In 2018, Chang (53) proposed that DIC should be reinterpreted according to “two-activation theory of the endothelium.” Infection, trauma, pathological obstetrics and other etiologies first activate the complement system and promote the generation of C5b-9 as a terminal membrane attack complex, resulting in endothelial injury and activation, and thus causing endotheliopathy. Subsequently, two important molecular events occur: activation of inflammatory pathway and microthrombotic pathway. The former triggers the release of cytokines, causing “inflammatory storm.” The latter mediates platelet activation and endothelial exocytosis of ultra-large von Willebrand factor (ULVWF), which is anchored to endothelial surface and recruits activated platelets to form microthrombus composed of platelet-ULVWF complexes. This process leads to thrombocytopenia and disseminated intravascular microthrombosis (DIT), resulting in MODS. Therefore, it is suggested that “DIC” should be redefined as “endotheliopathy-associated DIT.” This is a new understanding of the pathophysiological mechanism, but it still needs further verification.

### 6.2 The reestablishment of DIC diagnostic criteria

In order to establish reasonable DIC diagnostic criteria, the most important thing is to fully understand its pathophysiological mechanisms, such as the damaging effects and mechanisms of damage-associated molecular patterns (DAMPs) including histones (54), and the interaction between the pro-coagulant substances released after endothelial dysfunction, which remain to be further explored. It seems that it is imperative to redefine DIC based on different etiologies and pathophysiological mechanisms, such as “sepsis-induced coagulopathy,” “trauma-induced coagulopathy,” “cancer-induced coagulopathy,” “heatstroke-induced coagulopathy,” “pregnancy-related coagulopathy,” et al. Of course, personalized diagnostic criteria based on pathophysiology are also needed (55). The new DIC diagnostic criteria should have the following characteristics: sequential diagnostic criteria for early detection of DIC, the parameters included in the diagnostic criteria can reflect the understanding of pathophysiology, clear cut-off values of the parameters, clinical applicability, global unified (currently Japan and western countries tend to apply different diagnostic criteria) and be able to identify therapeutic patients. Since DIC is a complex syndrome caused by multiple etiologies, the combination of artificial intelligence stratification and the inclusion of sensitive biomarkers will help redefine DIC and identify phenotypes.

### 6.3 Early warning and stratification of DIC

Although the diagnosis of DIC is important, it is even more important to provide early warning of its onset and stratify DIC patients so that early intervention can be carried out in high-risk groups to prevent its progression. With the development of artificial intelligence, it may be the future direction to collect clinical information and biological samples, combine with bioinformatics to screen relevant biomarkers and identify the correlation with clinical information, so as to warn the onset of DIC or incorporate into diagnostic criteria, and then screen targeted patients for DIC anticoagulant therapy.

## 7 Conclusion

Disseminated intravascular coagulation, as a clinical syndrome caused by various etiologies, is complex in pathophysiology and highly heterogeneous. It is necessary to combine artificial intelligence with bioinformatics analysis to fully understand the pathophysiological mechanisms of various DIC, identify phenotypes and establish personalized diagnostic criteria, so as to provide precise targeted patients for anticoagulant treatment. The name and diagnostic criteria for DIC will be redefined in the near future.
